# Study on Crack Development of Concrete Lining with Insufficient Lining Thickness Based on CZM Method

**DOI:** 10.3390/ma14247862

**Published:** 2021-12-18

**Authors:** Jian Liu, Xuesen Zhang, Gaohang Lv, Kang Wang, Bo Han, Quanyi Xie

**Affiliations:** 1 School of Qilu Transportation, Shandong University, 12550 East Second Ring Road, Jinan 250002, China; liujianshanda@163.com (J.L.); sduqljtwk@163.com (K.W.); 2 School of Civil Engineering, Shandong University, 17922 Jingshi Road, Jinan 250061, China; lvgh1125@163.com (G.L.); bo.han@sdu.edu.cn (B.H.)

**Keywords:** tunnel, concrete, CZM, numerical simulation, insufficient thickness, crack mechanism

## Abstract

The most common structural defect of a tunnel in the operation period is the cracking of concrete lining. The insufficient thickness of tunnel lining is one of the main reasons for its cracking. This study studied the cracking behavior of standard concrete specimens and the failure behavior of tunnel structures caused by insufficient lining thickness using Cohesive Zone Model (CZM). Firstly, zero-thickness cohesive elements were globally inserted between solid elements of the standard concrete specimen model, and the crack development process of different concrete grades was compared. On this basis, a three-dimensional numerical model of the tunnel in the operation period was established. The mechanism and characteristics of crack propagation under different lining thicknesses were discussed. In addition, the statistics of cracks were made to discuss the development rules of lining cracks quantitatively. The results show that the CZM can reasonably simulate the fracture behavior of concrete. With the increase in concrete strength grade, the number of cohesive damaged elements and crack area increases. The insufficient lining thickness changes the lining stress distribution characteristics, reduces the lining structure’s overall safety, and leads to the cracking of the diseased area more easily. When surrounding rock does not contact the insufficient lining thickness, its influence on the structure is more evident than when surrounding rock fills the entire lining thickness. The number of cohesive damaged elements and the size of the crack area increases significantly.

## 1. Introduction

The tunnel was affected by different degrees of damage during operation. The most common structural damage was lining crack damage [1,2,3]. According to the rectification of tunnel defects, it was found that the main reason for tunnel defects was the lining defects caused by the construction process [4]. The lack of lining thickness is the most common defect form of the tunnel structure. The thinning of the lining is the weak point of the tunnel structure. Under the action of external force or water pressure, it is easy to cause a series of structural defects such as cracks and the leakage of the concrete lining structure of the tunnel, which seriously affect the safety of the tunnel structure and shorten its service life [5,6]. Therefore, it is of great significance to clarify the fracture behavior of the concrete lining of a typical tunnel.

The main reason for the disease of insufficient lining thickness was improper site construction, resulting in the insufficient excavation of surrounding rock [7]. Given the universality of insufficient lining thickness and its negative impact, domestic and foreign scholars pay increasing attention to it [8]. At present, scholars at home and abroad carried out some research on the concrete fracture behavior and the influence of defects, such as insufficient thickness, on tunnel operation safety by using a theoretical analysis, numerical simulation, on-site detection, and laboratory test, achieving abundant results [9,10,11,12].

Lackner and Amorim et al. established a calculation model to simulate the cracking of shotcrete and secondary lining concrete, respectively [13]. Zhang Chengping et al. studied the safety status of tunnel structure with a numerical simulation method due to insufficient lining thickness [14]. Based on the load-structure method, Wang Chunjing et al. established an evaluation model for the safety of supporting structures under the condition of insufficient lining thickness and evaluated the safety by the lining safety factor [15]. Wang Huarao et al. analyzed the influence of insufficient local lining thickness on internal force and the safety factor of the supporting structure under different surrounding rock conditions by using a finite element program; they also determined the safety state of the supporting structure accordingly [16]. Through indoor model tests, Chuan and Yu Jianqiu carried out static load failure tests on tunnel structure models under different stress fields and surrounding rock environment conditions, studied the structure failure law and ultimate bearing capacity, and gave corresponding reinforcement schemes for different defects [17,18]. Zheng Yangruong studied the propagation process of tunnel cracks with cavities behind the lining by using the extended finite element method [19]. Ansell investigated the existence of cavities behind the lining, and studied the distribution characteristics of cracks in the tunnel from the aspect of the water loss shrinkage of concrete in the secondary lining [20].

CZM is widely used to study the cracking of composites. Ahmad Amiri-Rad studied high cycle fatigue delamination in composite materials. CZM was used to simulate fatigue-driven delamination growth [21]. Ofir Shor and Reza Vaziri applied CZM to simulate two dynamic events involving the axial crushing of tubes and transverse impact loading of plates. The CZM numerical results were compared to the available experimental data [22]. Patryk Rozylo developed a cohesive element model to simulate the failure of composites caused by delamination. The experimental results agree well with the numerical results [23].

The mechanical properties of tunnel lining structures were widely studied. In this paper, the feasibility of CZM in ABAQUS, in order to simulate the fracture behavior of concrete, was verified by the simulation of the cracking behavior of standard concrete specimens. On this basis, the cracking mechanism of the concrete lining structure of highway tunnels with insufficient lining thickness was studied. The evolution process of cracks in tunnel lining structure was deeply analyzed. The influence mechanism of insufficient lining thickness on crack evolution of tunnel lining structure was obtained.

## 2. Study on Failure Mechanism and Crack Propagation of Concrete under Compression

**Figure 1:**
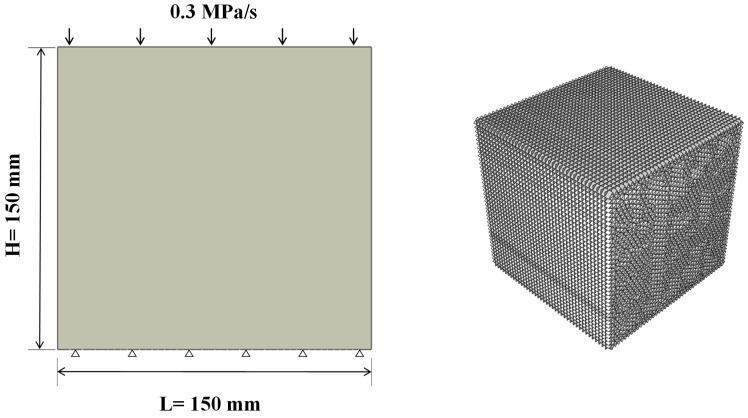
Numerical simulation of compressive strength of concrete.

In order to verify the feasibility of CZM to simulate the cracking behavior of concrete structures, a three-dimensional model with the same size as Testing Methods of Cement and Concrete for Highway Engineering was established in ABAQUS. The model size is 150 mm × 150 mm × 150 mm. A loading plate without thickness was set, endowed with an elastic modulus much higher than the specimens to ensure that the load could be uniformly applied on top of the specimen. The vertical displacement at the bottom of the model was fixed. The model and boundary conditions are shown in Figure 1. A continuous and uniform load was applied to the upper loading plate. According to the loading frequency of the concrete cube compressive strength test method in the Testing Methods of Cement and Concrete for Highway Engineering [24], the loading speed was 0.3 MPa/s, the same as that in the laboratory test.

For simulating the dynamic development of concrete cracks without preset paths, zero-thickness cohesion elements were embedded between solid elements to form potential crack surfaces [25,26,27]. In the calculation model, discrete solid elements include a continuum through zero-thickness cohesive elements. Each zero-thickness cohesive element’s top and bottom surfaces are connected with the solid elements and share nodes. In order to ensure the calculation accuracy, wedge element C3D6 is adopted for solid concrete elements, and COH3D6 and COH3D8 corresponding to wedge elements are adopted for cohesive elements. The model adopts fine mesh, and the total number of cells is 464,436. Simulation conditions and parameters are shown in Table 1 and Table 2.

### 2.1. Study on Crack Propagation of Concrete

**Figure 2:**
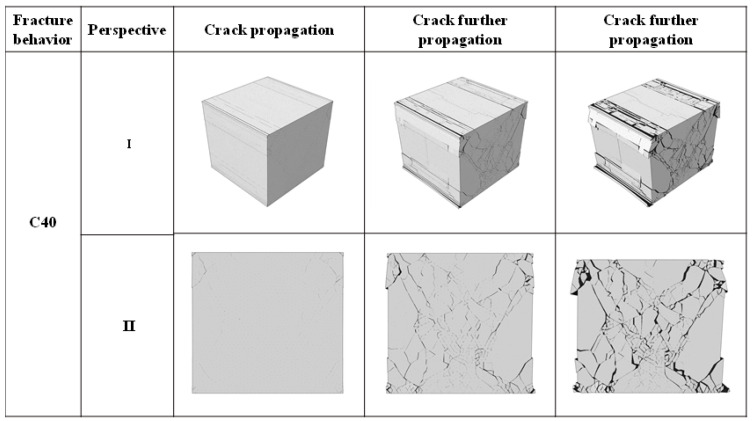
Fracture behavior of C40 concrete specimens under loading.

**Figure 3:**
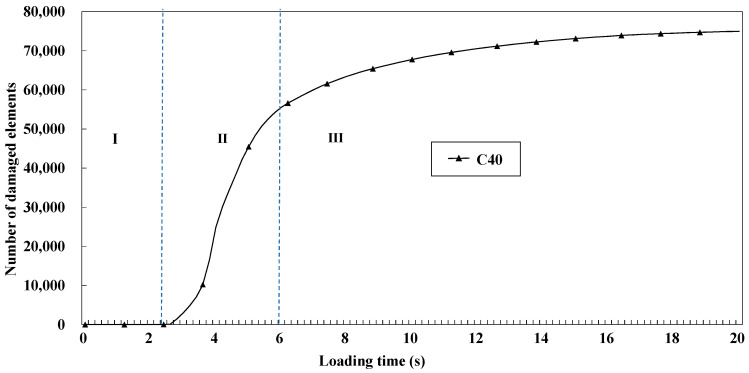
Number of cohesive damaged elements of concrete specimens.

This section took C40 concrete as an example for studying the cracking characteristics and crack development rules. In order to better observe the cracking of the model, we selected two perspectives, as shown in Figure 2. Perspective I is a 3D view showing the overall cracking of the concrete model, and the right-side view of perspective Ⅱ is a typical fracture surface. From perspective Ⅱ, it can be seen that the cracking first occurred at the corner of the concrete model and then developed rapidly to the center of the specimen along the direction of 45°, and the number of cracks increased rapidly. Finally, as the load increased, the width and volume of cracks increased significantly, but the number of cracks changed little. It also reflects the damaged number of cohesive elements of concrete specimens based on Python as shown in Figure 3. Combined with the cracking propagation of concrete in Figure 2, we can roughly divide the model’s failure into three stages. Stage I accumulated energy inside the concrete, and no cracks appeared in the model. In stage Ⅱ, as the load continued to increase, the damaged number of cohesive elements began to increase sharply. Macroscopically, cracks first appeared at the corners of the specimen, and then many cracks appeared with the increase in load. In stage Ⅲ, the damaged growth of cohesive elements slowed down and became stable, reaching 75,346 elements. Finally, several major macroscopic cracks converged in the microfracture area, and the phenomenon of collapse and shedding occurred at the edges and corners of specimens. In general, the crack pattern of concrete specimens under compression was significant, and similar results were obtained in Bo Wu’s uniaxial compression test of concrete [29].

**Figure 4:**
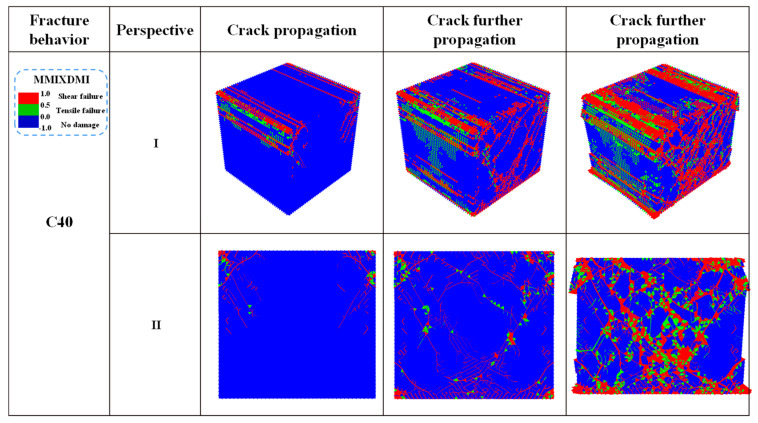
Damaged types of concrete cohesive elements: Overall distribution of the damaged cohesive elements and detailed cracking diagram.

In ABAQUS, the cracking mechanism can be identified by the damaged typed of cohesive elements (ABAQUS Manual) [25]. According to ABAQUS Manual, when the MMIXDMI value is in the range of 0–0.5, tensile damage predominates in the cohesive element. In contrast, when the MMIXDMI value is in the range of 0.5–1, shear damage predominates in the cohesive element. In addition, when the value is −1, the cohesive elements are not destroyed. Therefore, the MMIXDMI parameter is used to determine the cracking mechanism of the concrete model in this paper. Figure 4 selected the same perspective as Figure 2 to show the damaged types of cohesive elements at the crack growth stage. It can be seen from perspective Ⅱ that the red shear damage first appeared at the edge of the specimen, and then covered the whole view along the 45° direction. As the loading continued, the red shear damage range increased, and the green tensile damage was scattered next to the shear damage, which was also proven by the shear proportion of 70% in the model extracted based on Python. Compared with Figure 2, it can be seen that the damage rules of cohesive elements are consistent with the distribution of cracks.

### 2.2. Study on Cracking of Different Concrete Strength Grades

**Figure 5:**
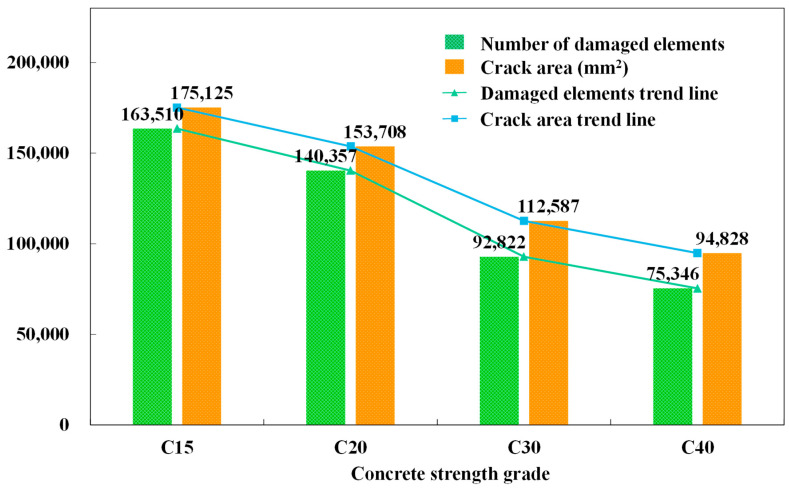
The number of damaged elements and crack area of different concrete strength grades.

**Figure 6:**
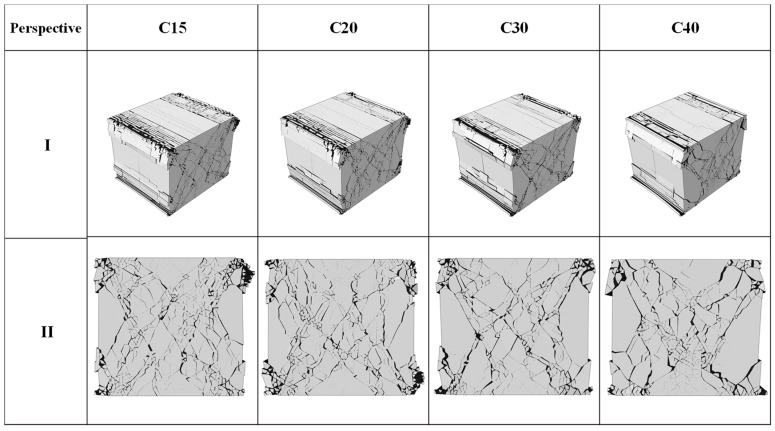
Crack distribution of different concrete strength grades.

This section analyzed the distribution form, crack area, and crack damage form of cracks under the same loading conditions by comparing the cracking of different strength grades of concrete: C15, C20, C30, and C40. According to Figure 5, under the same conditions, the number of damaged units of C15, C20, C30, and C40 is 163,510 and 1403, respectively. The crack areas are 175,125 mm^2^, 153,708 mm^2^, 112,587 mm^2^ and 94,828 mm^2^, respectively. As can be seen from the crack distribution in Figure 6, the crack area of the model increased with the increase in concrete strength. When the concrete strength is low, the phenomenon of edge shedding is more pronounced. In addition, it is worth noting that, with the increase in concrete strength, the maximum crack width increases, the number density of concrete surface cracks decreases. The ultimate crack width at C40 is 9.53 mm, an increase of 31% compared to C15. In compression, the higher the concrete strength grade is, the more likely small cracks are to develop into large oblique cracks. In terms of damaged fracture modes, the shear proportion of the four working conditions is 70%.

## 3. Study on Crack Propagation of Tunnel under Two Different Lining Thicknesses under Gradual Loading

### 3.1. Model Establishment

**Figure 7:**
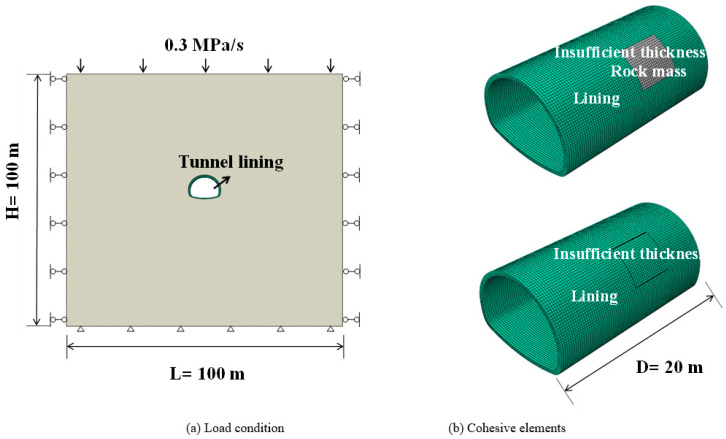
Numerical model and numerical elements.

The second section of this paper verified that CZM could be used to study the fracture of the concrete lining structure of highway tunnels. Therefore, this section uses CZM to study the cracking of lining structures under insufficient thickness of typical defects. The numerical model is shown in Figure 7a. The tunnel adopts the standard highway tunnel section, and the size of the surrounding rock is 100 m × 100 m × 20 m 3D model [27,30]. In terms of boundary conditions, the displacement of the surrounding rock on the left and right sides and the front and back directions was limited, and the vertical displacement at the bottom of the model was also fixed. A gradual loading of 0~10MPa was applied to the upper edge of the model, and the same loading frequency of 0.3 mpa/s was adopted as in Section 2 [31,32,33]. Wu also adopted similar loading conditions [34]. The surrounding rock element adopted C3D8R, the lining concrete parameter adopted C40, and the element set was the same as above. The parameters of related materials were shown in Table 3. Two conditions of insufficient lining thickness of different forms were selected for study, as shown in Figure 7b. The contact between the area with insufficient lining thickness and rock mass was mainly caused by under-excavation in the construction process [7]. The cavities with insufficient lining thickness mainly occur in the process of tunnel operation. This means that areas with an insufficient lining thickness do not contact the surrounding rock.

### 3.2. Fracture Behavior

**Figure 8:**
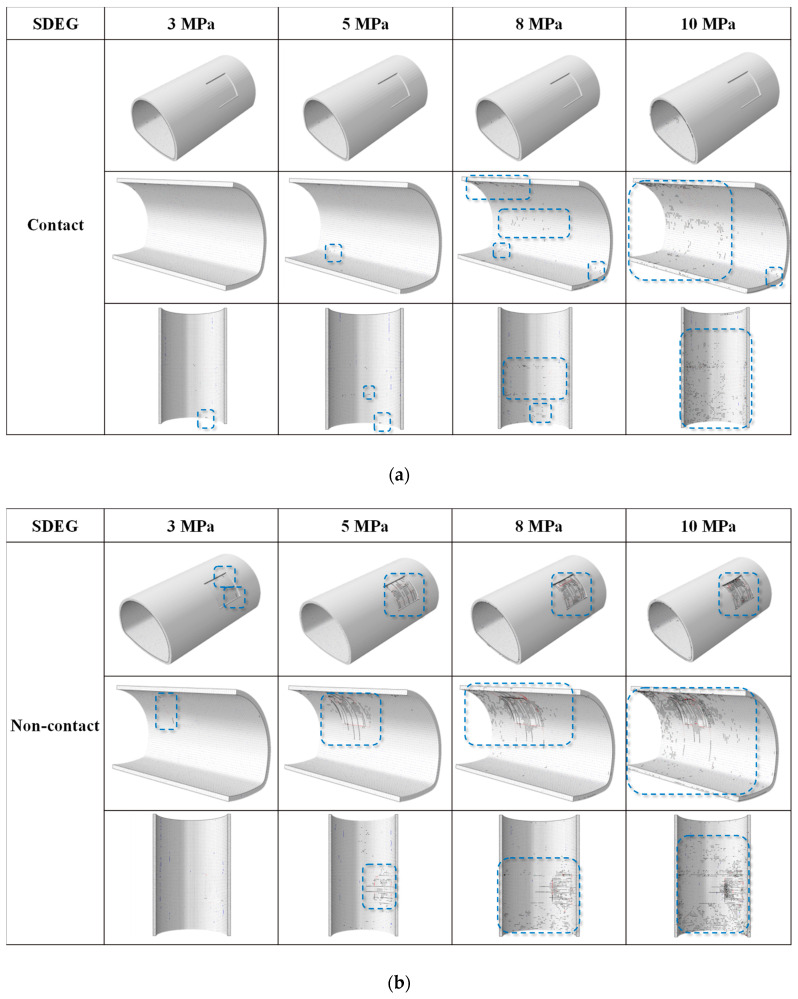
Fracture behavior of lining under gradual load. (**a**) Contact condition and (**b**) No-contact condition.

**Figure 9:**
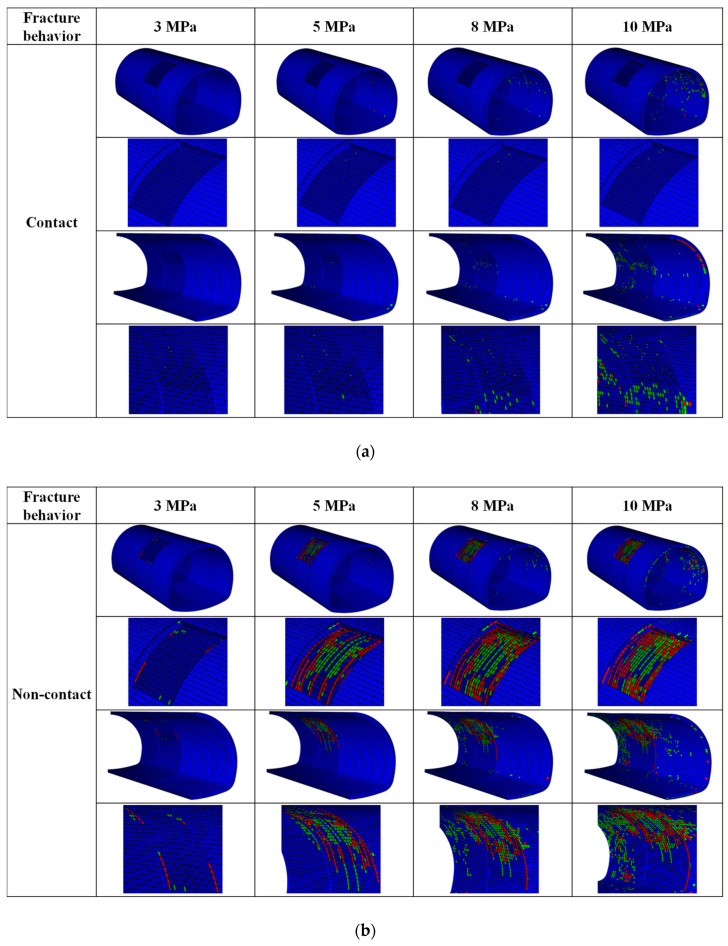
Damaged types of cohesive elements: Overall distribution of the damaged cohesive elements and detail cracking diagram. (**a**) Contact condition and (**b**) No-contact condition.

In this section, four values of 3 MPa, 5 MPa, 8 MPa, and 10 MPa are selected to discuss the characteristics of fracture and crack propagation under contact and non-contact conditions. As shown in Figure 8, we chose three typical perspectives to observe the phenomenon of lining cracking. As can be seen from the first perspective in Figure 8a, there is no crack on the outer surface of the lining under contact conditions. Perspective 2.3 in Figure 9a is the inner surface of the lining. As the load increases, the arch foot cracks first, followed by many circular cracks around the lining defects. Figure 8b shows the non-contact condition. As the load gradually increases, the number of cracks in the area with insufficient lining thickness increases. In addition, it should be noted that damages are mainly distributed in the lining defect area and are not apparent in other parts of the lining surface. As for the crack morphology produced in this study, we can find that the outer surface of the lining under non-contact conditions tends to produce ring cracks. The crack range increases with the increase in the applied load. At the same time, with the increase in vertical load at 5 MPa, longitudinal cracks appeared in the specimen, and the number of damages further increased. In addition, apparent ring cracks were observed on the inner surface of the lining defect. In the non-contact state, the lining mainly consists of circular cracks, and there are also longitudinal cracks. The inner surface of lining defects collapsed around 8 MPa. Overall, the insufficient lining thickness of crack mode was significant. The influence of the non-contact condition was significantly greater than that of the contact condition.

This section shows the damage types of cohesive elements in the propagation phase in Figure 9. Figure 9 shows the cracking mechanism of intricate parts, including the overall distribution of damaged adhesives and areas with an insufficient lining thickness. It can be seen that the cracking mechanism (damage type of cohesive elements) was significantly affected by insufficient lining thickness. Specifically, the failure behavior of cohesive elements in contact conditions was mainly tensile damage of the inner surface with insufficient lining thickness. In the non-contact condition, the shear damage of cohesive elements was distributed around the area of insufficient lining thickness, and tensile damage was the primary damage inside the area. In the non-contact condition, the tensile damage was caused by the tensile stress concentration on the outer surface. In addition, it was noticed that the edge of the insufficient lining thickness was mainly caused by shear failure. From the comparison of the two working conditions, it can be seen that the law of damaged viscosity elements was consistent within the distribution of cracks.

### 3.3. Statistics of Cracks

**Figure 10:**
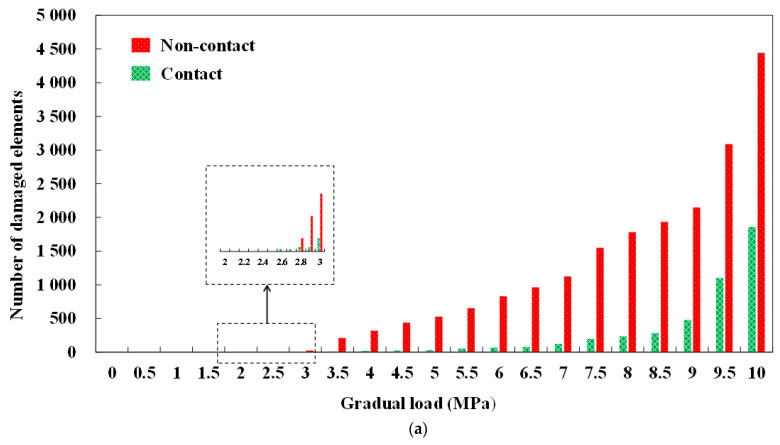

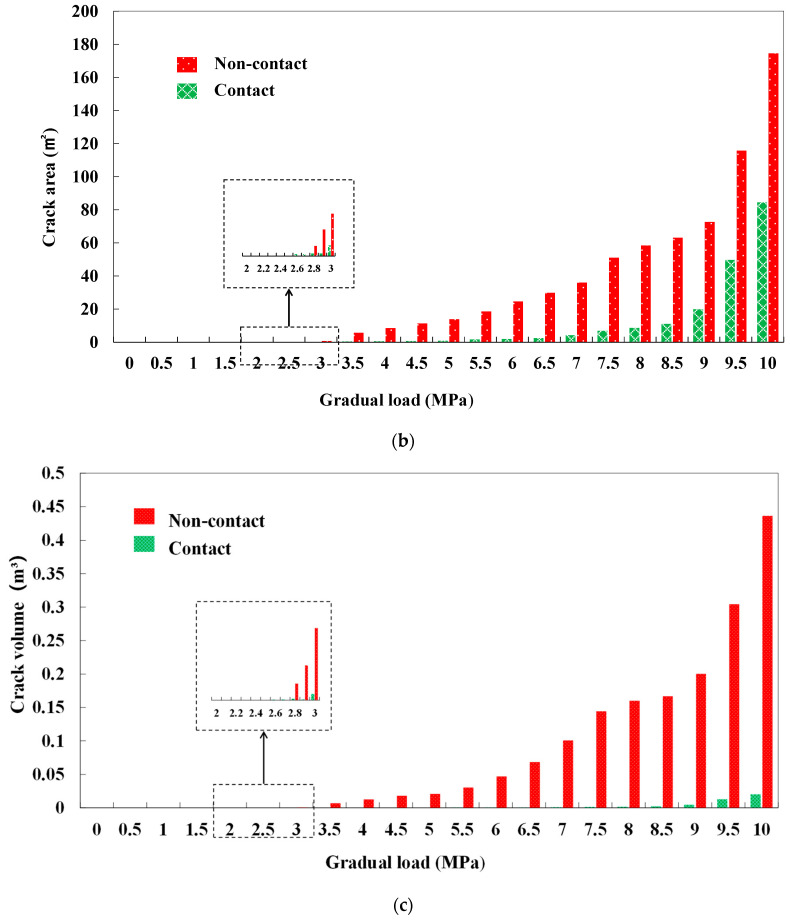

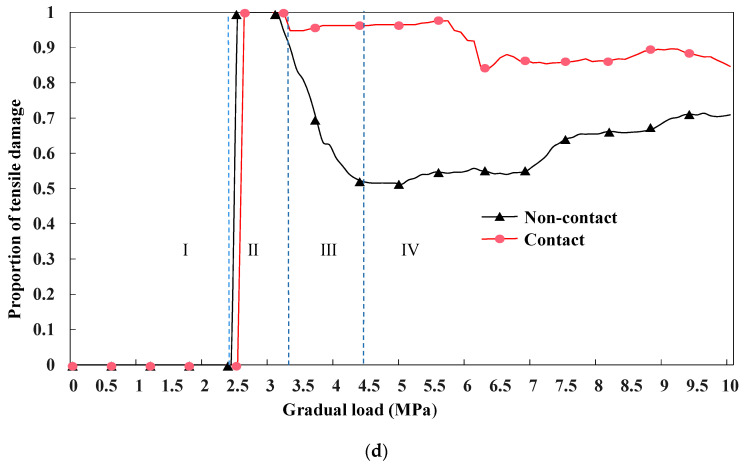
Statistics of cracks. (**a**) Number of cracking damaged elements. (**b**) Crack area. (**c**) Crack volume. (**d**) Proportion of tensile damage.

In order to clarify the characteristics of fracture evolution, the number of damaged cohesive elements, crack area, crack volume, and damaged form of cohesive elements were explored. Figure 10 shows the corresponding statistics. A number of damaged cohesive elements are shown in Figure 10a. When the incremental load was less than 3 MPa, the number of destroyed cohesive elements was small. With the further increase in the applied load, the number of damaged components increased significantly. Specifically, when the incremental load reached 10 MPa, 1857 damaged cohesive elements were cracked and damaged in contact conditions and 4443 were damaged in non-contact conditions. As for the distribution of lining fracture area and volume under gradual vertical loading, the output results are shown in Figure 10b,c.

With the increase in applied load, the crack area and volume further increase. In addition, it was also noted that the crack volume in non-contact conditions was much larger than that in contact conditions. From Figure 10d, it can be found that the proportion of tensile damage with the applied load can be divided into four stages, namely, crack-free stage Ⅰ (0–2.6 MPa), tensile damage stage Ⅱ (2.6–3.2 MPa), shear damage stage Ⅲ (3.2–4.5 MPa) and stable damage stage Ⅳ (4.5–10 MPa). In the crack-free stage, when the lining was subjected to the load transmitted by the surrounding rock, the cohesive elements begin to accumulate fracture energy but failed to reach the ultimate bearing capacity. At this time, the lining remained intact without cracks. As the load is further applied into the tensile damage stage, the damage of the cohesive elements are mainly tensile. In addition, according to the statistics of the number of damaged cohesive elements, the number of damaged cohesive elements at this time was minimal (less than 30 in both working conditions). At this time, the cracks are mainly small. With the increase in load, the crack is subjected to tensile damage and begins to enter the stage of shear damage. The proportion of shear damage elements begins to increase, and the proportion of tensile damage elements begins to decrease. According to the data, at 10 MPa, the tensile damage was 88.07% in contact and 60.28% in non-contact conditions. The shear damage was more remarkable in non-contact conditions.

## 4. Conclusions

In this paper, the CZM is used to study the cracking mechanism of highway tunnel lining structure under insufficient lining thickness. The main conclusions are as follows:

The feasibility of CZM in ABAQUS to simulate the fracture behavior of concrete was verified by the simulation of the cracking behavior of standard concrete specimens. Concrete is mainly subjected to shear failure in the compression process, which can be divided into three stages: stage I accumulates energy, stage II increases the number of cracks rapidly, and stage III increases the number of cracks slowly; small cracks gradually merge into large cracks. When the concrete strength grade is lower, the collapse of the edges and corners of concrete specimens are more pronounced. As the increase in concrete intensity grade, concrete crack area, and maximum crack width increase, long crack quantity increases and concrete surface crack quantity density decreases.

When the lining thickness was insufficient and did not contact the surrounding rock, cracks began to appear when the load increased to about 3 MP under gradual loading. With the increase in load, the area of circumferential cracks increased gradually, and the radial cracks appeared and developed. The damaged form of cracks changes from tensile damage to shear damage. The number of cohesive elements damaged, in general, increases rapidly, and the collapse phenomenon occurs on the inner surface of the lining with insufficient thickness. When the wall rock is full of the entire lining, thickness is insufficient. With the increase in load, there is almost no external surface of cracks. A large number of disordered short circumferential cracks were distributed on the inner surface. The damaged form is mainly tensile damage. In general, insufficient lining thickness significantly influences the cracking of concrete lining structures, and the impact of non-contact conditions is substantially more effective than that of contact conditions.

## Figures and Tables

**Table 1:** Numerical simulation conditions and concrete material parameters [24].

Conditions	Concrete Strength Grade	Density ρ (kg/m^3^)	Elasticity Modulus E (MPa)	Poisson’s Ratio *v*
Condition 1	C15	2360	22,000	0.20
Condition 2	C20	2370	25,500	0.20
Condition 3	C30	2390	30,000	0.20
Condition 4	C40	2400	32,500	0.20

**Table 2:** Parameters of cohesive elements materials [28].

Concrete Strength Grade	Density ρ (kg/m^3^)	Normal Traction (MPa)	Tangential Traction (MPa)	Fracture Energy/(N/m)	
C15	2360	5	16	55	120
C20	2370	5.5	18	65	135
C30	2390	6.5	22	80	185
C40	2400	8	30	100	200

**Table 3:** Parameters of rock mass and tunnel lining materials.

Part	Density ρ (kg/m^3^)	Elasticity Modulus E (GPa)	Poisson’s Ratio v	Cohesion c (kPa)	Internal Friction Angle φ (°)
Rock mass	1800	1.5	0.35	150	20
Lining solid elements	2390	30	0.2	-	-

## Data Availability

Some or all data, models, or code that support the findings of this study are available from the corresponding author upon reasonable request.
